# Editorial: Role of protein palmitoylation in synaptic plasticity and neuronal differentiation, volume II

**DOI:** 10.3389/fnsyn.2024.1473989

**Published:** 2024-09-10

**Authors:** Kevin P. Koster, William N. Green

**Affiliations:** Department of Neurobiology, University of Chicago, Chicago, IL, United States

**Keywords:** palmitoylation, depalmitoylation, synaptic transmission, synaptic plasticity, post-translational modification

Post-translational modifications (PTMs), such as *S-*palmitoylation described here, add to the already large diversity of protein amino acid code. These modifications act as a dynamic switch that regulates protein function by altering protein localization and activity, as well as interactions with other molecules, lipids, glycans, and nucleic acids. Most PTMs utilize charged or polar groups as the covalent modifications that trigger this switch. Protein *S-*palmitoylation is one of a group of PTMs that differs from most others in that a hydrophobic, lipid-like group is linked to the substrate during the modification. For *S-*palmitoylation, usually the fatty acid palmitate is the added modification. As a result, the modification causes cytoplasmic proteins to be first targeted to and then to associate with the cytoplasmic leaflet of the plasma membrane or other organellar membranes. Integral membrane proteins are also *S-*palmitoylated. However, the role of *S-*palmitoylation of membrane proteins is more subtle than for cytoplasmic proteins, aiding in the sorting of proteins to specialized lipid domains such as lipid rafts (see Hayashi article below) or the selective trafficking between endomembranes along the secretory pathway (Smotrys and Linder, 2004; Aicart-Ramos et al., 2011).

The most common type of palmitoylation, *S-*palmitoylation, is reversible, occurs via thioester linkage to cysteine residues, and is catalyzed by palmitoyl acyltransferases (PATs). This is distinct from other forms of protein palmitoylation (e.g., *N-*palmityolation), which are irreversible and use a different set of palmitoylating enzymes (Linder and Deschenes, 2007; Resh, 2021). PATs are large, multi-pass integral membrane proteins containing a conserved catalytic site positioned near the inner membrane leaflet (Fukata et al., 2004; Greaves and Chamberlain, 2011; Korycka et al., 2012). This conserved region consists of the aspartate-histidine-histidine-cysteine (DHCC) sequence, and thus, PATs are also referred to as zDHHC proteins (Fukata et al., 2004; Greaves and Chamberlain, 2011; Korycka et al., 2012). Considering *S-*palmitoylation is typically a dynamic and reversible process, both the palmitoylation and depalmitoylation processes act to regulate modified proteins. Depalmitolyation is mediated by cytoplasmic thioesterases which are still being characterized (Lin and Conibear, 2015a,b; Won et al., 2018; Koster and Yoshii, 2019).

The importance of *S-*palmitoylation is evident, since this modification occurs on ~11% of the human proteome (Blanc et al., 2015). This modification and other PTMs are highly prevalent in the proteome (Kang et al., 2008; Sanders et al., 2015), as their dynamic nature coincides with the shuttling of molecules (or groups of molecules) to and from synaptic domain being the basis for plasticity. In the nearly 5 years since the first volume of this Research Topic was published (Yoshii and Green, 2020), more progress has been made in characterizing how protein *S*-palmitoylation influences synaptic form and function, neuronal plasticity, and the brain at the systems level. Much of the most recent work centers on the influence of synaptic activity/plasticity on synaptic protein palmitoylation, building on the seminal findings of Noritake et al. (2009). For instance, several new studies demonstrate activity-dependent changes to synaptic protein palmitoylation, including the enzymes that mediate palmitoylation themselves (Nasseri et al., 2022; Abazari et al., 2023). Similarly, multiple lines of experiments (Shen et al., 2022; Koster et al., 2023) reveal that the palmitoylation of postsynaptic scaffolds, neurotransmitter receptors, and other synaptic molecules are crucial for mediating homeostatic synaptic plasticity (Turrigiano et al., 1998; Turrigiano, 1999). Finally, recent work also solidifies a role for synaptic protein palmitoylation in various forms of plasticity that may underlie learning across different species (Nelson et al., 2020; Nasseri et al., 2022; Seo et al., 2022). In the current Research Topic, several groups have examined how palmitoylation of synaptic proteins affects their precise localization, interactions with other synaptic molecules, and the downstream consequences of dysregulated synaptic palmitoylation.

In a brief article reporting new findings with respect to long-term plasticity at glutamatergic synapses, the Hayashi lab presents new evidence that phosphorylation of the GluA2 subunit of AMPA receptor at tyrosine 876 (GluA2-pTyr876) requires intact lipid rafts. Specifically, Hayashi demonstrates that Tyr876 phosphorylated GluA2 is largely localized to a biochemically isolated lipid raft fraction in primary cortical neurons. Further, disruption of lipid rafts abolished the typical stimulation-dependent phosphorylation of GluA2 at Tyr827. Together, these results suggest a crucial role for palmitoylation of Src member kinases like Fyn in the selective trafficking of GluA2 to lipid rafts, providing a model by which palmitoylation controls the compartmentalization of kinases to regulate synaptic strength.

A study from Barylko et al., examines the palmitoylation-dependent localization of calmodulin kinase-like vesicle-associated (CaMKv), a pseudokinase that is required for the maintenance of dendritic spines and typical synaptic physiology (Liang et al., 2016). The authors demonstrate that CaMKv is indeed palmitoylated at the predicted site (cysteine 5; Collins et al., 2017) and that its palmitoylation is required for localization of CaMKv to the plasma membrane. Intriguingly, the article also shows that CaMKv directly interacts with the immediate early gene, Arc, in a palmitoylation-dependent fashion. These data suggest that the palmitoylation of both Arc and CaMKv are involved in the activity-dependent endo- and exocytosis of AMPA receptors during Hebbian plasticity.

The studies from Chen et al., and the Yoshii lab group both provide novel insight into how palmitoylation of the A-kinase anchoring protein 5 (Akap5) influences its role as a postsynaptic scaffold. First, a detailed light and electron microscopic study of Akap5 nanoscale organization demonstrates that N-terminal palmitoylation of Akap5 is necessary for it adapting a vertical (i.e., extended) orientation relative to the postsynaptic membrane, similarly to PSD-95 (Jeyifous et al., 2016). Palmitoylated Akap5 is also more likely to associate with the postsynaptic density and synaptic endosomes, while palmitoylation had a lesser effect on the extrasynaptic Akap5 pool. Performing the same analyses with a palmitoylation-deficient mutant of Akap5 revealed that concurrent with loss of this characteristic vertical, extended orientation, it is displaced from the postsynaptic density. These findings indicate that palmitoylation of Akap5 underpins its stability in synaptic sub-compartments, like the postsynaptic density.

In a series of experiments, the lab of Dr. Akira Yoshii demonstrates that Akap5 is overly palmitoylated in a model of infantile Batten disease, which occurs due to a mutation in a depalmitoylating enzyme. Building on the evidence from Chen et al. here, as well as previous studies on Akap5 function (Purkey et al., 2018; Sanderson et al., 2018), they demonstrate that the excessive palmitoylation of Akap5 in this disease model correlates with a plasticity-dependent excessive upregulation of the AMPA receptor subunit GluA1. Further scrutiny implied that aberrant Akap5 palmitoylation links synaptic receptor activation to downstream pro-inflammatory signaling through its transcriptional targets, which exacerbated the Batten disease phenotype under these circumstances. Taken together, these studies point to the palmitoylation state of Akap5 as a critical regulator of glutamate receptors and local signaling pathways.
